# The effect of perceived parental rejection on depression in high school students: the chain mediating role of rumination and alexithymia

**DOI:** 10.3389/fpsyg.2025.1666593

**Published:** 2026-01-12

**Authors:** Xiangyan Li, Quan Lu, Yuxin Wang, Yuan Gao

**Affiliations:** 1 Faculty of Education, Henan Normal University, Xinxiang, China; 2 Zhengzhou Educational Science Planning and Evaluation Center, Zhengzhou, China; 3 School of Psychology, South China Normal University, Guangzhou, China

**Keywords:** perceived parental rejection, depression, rumination, alexithymia, high school students

## Abstract

In recent years, the incidence of depression in adolescents has continued to rise, which has become a hot issue of global concern. Through a cross-sectional survey, this study aims to explore the relationship between perceived parental rejection and depression in high school students, and to test the chain mediating effect of rumination and alexithymia in the relationship between them. A questionnaire survey was conducted among 2,310 high school students using the Short-from Egna Minnen av Barndoms Uppfostran for Chinese Scale, the Ruminative Responses Scale, the Toronto Alexithymia Scale and the Chinese version of Beck Depression Inventory-II (BDI-II). The results showed that parental rejection was positively correlated with depression in high school students. The mediating effects of rumination and alexithymia between parental rejection and depression were significant. Rumination and alexithymia played a chain mediating role between parental rejection and depression. Conclusion: Parental rejection not only directly predicts the depression levels of high school students, but also indirectly affects their depression through the independent mediating effects of rumination and alexithymia, as well as the chain mediating effect of rumination-alexithymia. This study constructs and validates this model for the first time, thereby deepening the understanding of the complex mechanism between parental rejection and depression in high school students and providing important insights for the prevention and intervention of depression.

## 1 Introduction

Depression is the leading cause of disability worldwide (Dhingra et al., 2023) and one of the most dangerous mental illnesses threatening the physical and mental health of adolescents (Mao et al., 2023). It is primarily characterized by sadness, loss of interest or pleasure, feelings of guilt or low self-worth, sleep and appetite disturbances, fatigue, and inattention (World Health Organization, 2017). Approximately 8% of adolescents globally are affected by depression (Madigan et al., 2023; Shorey et al., 2022). Research indicates that depression can originate in childhood and significantly increase in prevalence during adolescence (Korczak et al., 2023; Morken et al., 2021). Adolescence is a high-incidence period for depression, particularly among high school students. These students are in a phase of psychological conflict between identity formation and role confusion, making them susceptible to emotional fluctuations and various psychological issues (Blankenstein et al., 2020; Zou et al., 2020). Additionally, high school students face multiple adverse factors, including interpersonal stress, academic burden, and social changes, which contribute to significant psychological pressure (Kulakow et al., 2021) and more severe depressive symptoms. Depression has numerous negative impacts on high school students, interfering with daily functioning and leading to adverse outcomes such as decreased academic performance (Liu et al., 2018) and social withdrawal (Yang et al., 2024). In severe cases, it can result in critical events such as self-harm and suicide (Fitzpatrick et al., 2023; Kalin, 2021). Therefore, exploring the mechanisms underlying depression is of great practical significance for the effective prevention and intervention of depression among high school students.

As the primary microsystem for adolescent development, the family's parenting style and the quality of parent-child interaction are key antecedent factors influencing an individual's mental health (Yang et al., 2024). Among these, perceived parental rejection, as a negative parent-child interaction experience, has been confirmed to be closely associated with adolescent depression (Yu et al., 2024). However, most existing studies have only focused on exploring the direct correlation between parental rejection and adolescent depression, and there is still a lack of systematic and in-depth exploration of the internal mechanism underlying how the two are linked.

The COVID-19 pandemic has exerted adverse effects on adolescents' mental health, leading to symptoms such as depression, anxiety, and rumination (Liu et al., 2022). Studies have indicated that maladaptive parenting practices may act as an inducer of individuals' rumination (Meng et al., 2024). Adolescents who perceive parental rejection exhibit significantly elevated levels of ruminative thinking. Such persistent rumination continuously reinforces their negative cognition, thereby increasing the risk of developing depression (Schweizer et al., 2018).

As the primary context for emotional socialization, the family plays a crucial role in shaping children's emotional functioning through parental emotional responsiveness and communication patterns (Cooke et al., 2019). Chronic perceived parental rejection may hinder the fulfillment of emotional expression and recognition needs, predisposing individuals to develop alexithymia (Lukas et al., 2022). Concurrently, alexithymia impairs the effective regulation of negative emotions, further contributing to the onset of depression (Hemming et al., 2019).

Rumination and alexithymia have long been regarded as mutually influential constructs (Yuan et al., 2025). Existing studies have verified the independent roles of rumination and alexithymia as risk factors for depression (Mo et al., 2025; Lu et al., 2024). Nevertheless, previous research has not established the interaction between cognitive mechanisms (ruminative thinking) and affective mechanisms (alexithymia) to interpret the association between “parental rejection and depression among senior high school students.” This makes it difficult to clearly elucidate the complete pathway and internal logic through which negative family factors are transformed into individual psychological deficits. Therefore, the main purpose of this study is to explore the relationship between perceived parental rejection and depression in senior high school students via questionnaire surveys, and to focus on examining the influence pathways of ruminative thinking and alexithymia between the two variables.

More specifically, this study aims to address the following research questions:

RQ1 Can perceived parental rejection positively predict depression in adolescents?

RQ2 What roles do ruminative thinking and alexithymia play in the relationship between perceived parental rejection and adolescent depression?

### 1.1 Theoretical foundation

The Process Model of Emotion Regulation proposed by Gross (1998), which centers on the “dynamic process of emotion generation and regulation,” divides emotion regulation into five consecutive stages: “situation selection, situation modification, attention deployment, cognitive change, and response modulation.” This model provides theoretical support for interpreting the complex pathway of how parental rejection influences adolescent depression. Specifically, when high school students encounter the negative situational stimulus of parental rejection, individuals with a high tendency for rumination are prone to regulatory biases in the “cognitive change” stage. Their thinking fails to focus on constructive, problem-solving-oriented cognition; instead, they fall into repetitive rumination about negative experiences. This cognitive fixation not only amplifies the experience of negative emotions but also hinders the smooth transition of emotional processing to subsequent stages, laying a cognitive foundation for the development of alexithymia. Eventually, the entire “situation-cognition-response” chain in the Process Model of Emotion Regulation suffers a functional breakdown. Negative emotions cannot be alleviated through effective regulation and thus accumulate continuously, which in turn induces depressive symptoms.

### 1.2 The relationship between perceived parental rejection and depression

Perceived parental rejection is defined as an adolescent's belief that his or her parents are not concerned or interested in him or her as a person (Robertson and Simons, 1989), parents wanting the adolescent to be a different person or parents frequently criticizing the adolescent (Muris et al., 2001). The Chinese National Mental Health Development Report (2023-2024) provides an in-depth analysis of the mental health status and its influencing factors. The survey indicates that family dysfunction, including parental absence, parental rejection, excessive control, and emotional neglect, is highly correlated with the risk of depression. Studies have confirmed that adolescents who frequently perceive parental rejection are more likely to experience depression (Pachankis et al., 2018). Parental rejection has a devastating impact on adolescents, as the parent-child relationship is particularly complex during this period (Brown and Bakken, 2011). According to the social measurement theory, individuals possess an internal monitoring system that assesses and responds to the degree of acceptance or rejection they encounter in social interactions (Leary, 2005). The experience of being accepted or rejected significantly affects an individual's psychological state and behavior. The family is one of the most direct and important micro-environments influencing the psychological development of adolescents (Bronfenbrenner, 1979), and the influence of parents on adolescents is substantial. The interpersonal acceptance-rejection theory proposed by Rohner (2021) provides a theoretical framework for understanding the impact of perceived parental rejection on the emotions, cognition, and behavior of adolescents. The theory identifies four manifestations of parental rejection: (1) emotional indifference and lack of care, (2) hostility and aggression, (3) neglect and disregard of children's needs, and (4) unconditional rejection. Individuals who perceive parental rejection may experience a lack of self-worth (Sethi, 2024), leading to the development of maladaptive personality traits and an increased likelihood of exhibiting externalized behavioral problems (e.g., alcoholism, drug abuse, aggressive behavior) and internalized emotional problems (e.g., anxiety, depression). Therefore, this study proposes the following hypotheses:

H1: There is a positive correlation between perceived parental rejection and depression.

### 1.3 The relationship between perceived parental rejection, rumination, and depression

Rumination refers to the continuous focus on negative experiences following adverse life events, the persistent contemplation of the causes and consequences of these events, and the associated negative emotions, rather than actively seeking solutions to problems (Nolen-Hoeksema, 1991). As an automated coping strategy, rumination is unconsciously employed by individuals to manage painful experiences (Smith and Alloy, 2009). However, this mode of thinking does not process emotions but instead functions as an unhealthy coping mechanism by fostering negative self-thoughts (Samtani and Moulds, 2017). According to the Response Styles Theory, rumination can influence individuals' susceptibility to depression, the severity of symptoms, and their duration (Nolen-Hoeksema, 1991). This mode of thinking amplifies the perceived importance of stressors, leading to excessive and distorted cognitions of problems and, consequently, exacerbating negative emotional states (Nolen-Hoeksema, 1991). Beck's Cognitive Model of Depression posits that depression stems from negative, pessimistic, and irrational thinking (Leahy, 2002). Rumination, as a maladaptive strategy, may induce depression or contribute to its recurrence (Brakowski et al., 2017; LeMoult and Gotlib, 2019). Previous studies have demonstrated that rumination is a significant indicator of various negative physical and psychological outcomes, including depression and anxiety (DeJong et al., 2016; Liang et al., 2020), and that a persistent correlation exists between rumination and depression (Raffaelli et al., 2021). Because rumination prompts adolescents to repeatedly review painful events, their potential consequences, negative emotional experiences, and negative self and environmental evaluations, it promotes the formation and consolidation of the link between negative emotions and cognitive processes, thereby increasing the level of depression in adolescents (Ji, 2024).

Children's socialization, internalization, and externalization are highly susceptible to the interdependence among family members, particularly their interactions with parents. Research has demonstrated that negative parenting styles may prompt children to adopt negative thinking patterns when confronted with difficulties and challenges, potentially leading to rumination (Lionetti et al., 2022). A correlation mechanism exists between parental rejection and rumination, as parental rejection can impair children's cognitive and emotional functioning (Quirk et al., 2015). According to the parental rejection-acceptance theory, dysfunction arises from the rejection of children's support needs, causing them to internalize unsafe processing patterns, which manifest as alienation from parents and heightened self-anxiety (Rohner et al., 2012). Based on these findings, rumination may serve as an individual difference risk factor linking parental rejection to adolescent depression. Therefore, this study proposes the following hypotheses:

H2: Rumination plays a mediating role between parental rejection and depression.

### 1.4 The relationship between perceived parental rejection, alexithymia, and depression

Alexithymia, also known as the inability to express emotions, refers specifically to an individual's lack of ability to describe and recognize their own and others' emotions, as well as the presence of limited imagination and externally oriented thinking (Sifneos, 1973). It was initially discovered by psychiatrist and psychological counselor Sifneos (1973) among patients with physical and mental disabilities. Later investigations showed that in non-clinical groups, the prevalence of alexithymia usually ranged from 7.3 to 29.9% (Honkalampi et al., 2009). Therefore, alexithymia is now widely recognized as a personality trait within the field of psychology (Lyvers et al., 2022; van der Velde et al., 2015). According to the Family Systems Theory (Bowen, 1993), family cohesion fosters emotional interconnectedness and behavioral correspondence among family members, highlighting the significant role of the family environment in the development of alexithymia in children. Parental rejection, a negative parenting approach, has been shown to significantly predict children's psychopathological symptoms, particularly emotional symptoms (Giaouzi and Giovazolias, 2015; Sart et al., 2016), as well as emotional regulation difficulties (Casselman and McKenzie, 2015; Mendo-Lázaro et al., 2019). Research by Hussain and Ahmed (2014) indicated that perceived parental rejection significantly predicted the occurrence of alexithymia in adolescents. Parental rejection reduced emotional expression in both adolescents and parents within the family environment, negatively impacting the development of adolescents' emotional functioning and subsequently inducing alexithymia (Ng and Chan, 2020).

Relevant studies have demonstrated that alexithymia is highly associated with depression, anxiety, and stress (Preece et al., 2024) and significantly increases the risk of depression in adolescents (Tang et al., 2022). Alexithymia may increase the risk of depression through multiple pathways. Firstly, individuals with alexithymia experience difficulties in recognizing and expressing their emotions, as well as in obtaining effective social support and emotional understanding. These difficulties hinder their ability to effectively cope with emotional stimuli or stressors, leading to emotional suppression, which manifests as persistent anxiety and restlessness. The accumulation of these negative emotions can eventually evolve into depression (Desdentado et al., 2023). Secondly, individuals with alexithymia face challenges in both the attention allocation and evaluation stages of emotion processing (Preece and Gross, 2023) and are unable to conduct effective emotion regulation. Thus, it is accompanied by a variety of internalized symptoms, such as anxiety and depression (Prino et al., 2019; Honkalampi et al., 2000; Qian et al., 2020). Finally, individuals with alexithymia typically lack effective emotion regulation strategies, making them more likely to feel helpless and hopeless when confronted with life challenges. Their inability to obtain effective social support and emotional understanding exacerbates feelings of loneliness and low self-esteem, thereby increasing the risk of depression (Sun et al., 2024). Based on this, we propose the following hypotheses:

H3: Alexithymia plays a mediating role between parental rejection and depression.

### 1.5 The chain mediating effect of rumination and alexithymia

In a study on depression, patients with alexithymia were found to exhibit significantly higher levels of rumination compared to non-alexithymic individuals (Du and Dong, 2019). This finding corroborates the significant correlation between rumination and alexithymia. Subsequent research further identified a positive correlation between rumination and alexithymia (Yildirim Ayaz and Dincer, 2021). Rumination is defined as an individual's ineffective cognitive control of negative emotions, while alexithymia is mainly manifested in the inability of individuals to recognize their own and others' emotions, and the lack of emotional expression ability. There is an internal consistency between them (Zhang et al., 2011). Research has shown that rumination is an antecedent of alexithymia and exerts a significant predictive effect on its onset (Bal and Alkoç, 2020). According to the Cognitive-Emotional Processing Model, long-term rumination impairs emotional regulation ability and amplifies emotional distress through dual cognitive-affective pathways. It is indirectly associated with alexithymia, forming a vicious cycle characterized by “cognitive fixation-emotional blunting” (Yuan et al., 2025). When exposed to negative stimuli, individuals with high trait rumination demonstrate a tendency to become immersed in negative affective states. Frequent use of suppressing emotions to regulate negative emotions may lead individuals to develop negative self-evaluations and increase their risk of emotional disorders (Colzato et al., 2020). Studies have also shown that rumination as a kind of negative response style, can significantly influence an individual's emotional expression (Feldman et al., 2008). Individuals with higher levels of rumination may struggle to accurately identify positive emotions in themselves and others (Ning et al., 2015), which could lead to the emergence of alexithymia symptoms (Zhang et al., 2011). Therefore, this study proposes the following hypotheses:

H4: Rumination and alexithymia play a chain mediating role between parental rejection and depression.

**Figure 1:**
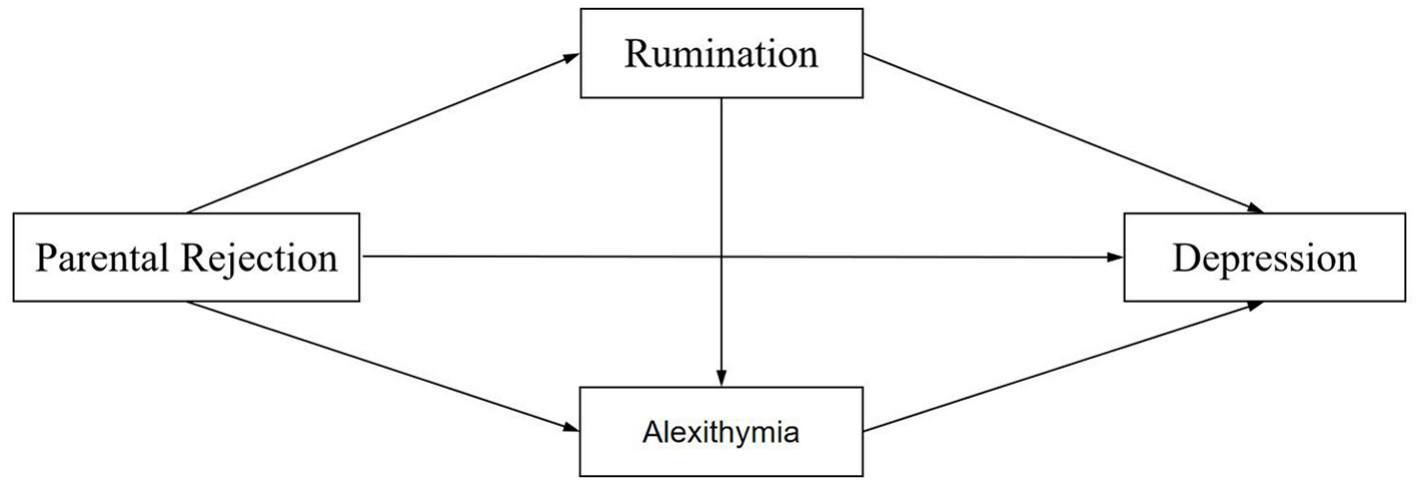
Schematic diagram of model.

Based on the above literature review and research hypotheses, this study, from the theoretical perspective of the Process Model of Emotion Regulation, constructs a chain mediating model (as shown in Figure 1). Its core lies in focusing on the interaction between cognitive and emotional mechanisms, and establishing an integrated “family-cognition-individual” multi-system model. From the dual perspectives of cognitive-emotional processing, this study deepens the understanding of the internal mechanism underlying the relationship between parental rejection and adolescent depression. It can not only expand the application scenarios of emotion regulation theory in adolescent depression research and improve the theoretical system of depression pathogenesis, but also provide practical references for family parenting guidance, school mental health education, and clinical psychological interventions.

## 2 Method

### 2.1 Participants

The participants were recruited from two high schools in Henan Province, China, and the sample comprised 2,310 high school students. Specifically, 1,732 students were in their first year of high school, and 578 were in their second year. The sample also included 1212 boys and 1098 girls. The gender distribution of participants by grade is presented in Table 1. The adolescents in the sample ranged in age from 14 to 19 years, with a mean age of 15.88 years (SD = 0.86).

**Table 1:** Descriptive statistics of the participants.

**Grade**	**Gender**		**Total**
		**Male**		**Female**	
Grade 10	908 (52.4%)	824 (47.6%)	1732
Grade 11	304 (52.6%)	274 (47.4%)	578
Total	1212 (52.5%)	1098 (47.5%)	2310

### 2.2 Procedure

The study employed a convenient cluster sampling method and was conducted at two high schools in Henan Province, China. Approval was obtained from the school administration after clearly explaining the research plan. The research team informed parents and students about the nature of the survey through the head teacher and obtained informed consent from parents, as well as signatures from the students themselves. All participants were clearly advised that they had the right to withdraw at any time and that their information would be kept confidential, with the collected data being used solely for academic research purposes. Standardized paper questionnaires were used for data collection. The implementers, comprising graduate students and teachers who had undergone unified training, were assigned to standardize the instructions and points of attention during the process. To ensure data authenticity, strict supervision was carried out throughout the investigation. Upon completion of the survey, the researchers collected the questionnaires and entered the data.

### 2.3 Measurements

#### 2.3.1 Parental rejection scale

In this study, perceived parental rejection was assessed using the Short-from Egna Minnen av Barndoms Uppfostran for Chinese Scale (S-EMBU-C) (Jiang et al., 2010). The scale consists of 42 items and is divided into two parts: father and mother. The parental rejection dimension includes items 1, 4, 7, 13, 16, and 21, which are rated on a 4-point Likert scale. The average of the scores from the two parts on parental rejection represents the score for parental rejection. A higher score indicates a more pronounced tendency toward parental rejection in parenting style (Sun et al., 2024; Yu et al., 2024).

#### 2.3.2 Ruminative responses scale

In this study, the Chinese version of the Ruminative Response Scale (RRS) used was translated and revised by Han and Yang (2009). The RRS consists of 22 items, including three dimensions: symptomatic ruminative, compulsion thinking and introspection thinking. Participants responded to the questions on a 4-point Likert scale, ranging from 1 (never) to 4 (always). The total score for each participant was calculated by summing the scores of all items. Higher scores indicate a greater degree of rumination.

#### 2.3.3 Toronto alexithymia scale

The Toronto Alexithymia Scale (TAS) used in this study was compiled by Bagby et al., and revised by Yi et al. (2003). The TAS is a self-report questionnaire containing 20 items on a 5-point Likert scale, ranging from 1 (strongly disagree) to 5 (strongly agree). The scale included 3 subscales: difficulty describing feelings (5 items), difficulty identifying feelings (7 items), and externally-oriented Thinking (8 items). The questionnaire contains 5 reverse-scored items (numbers 4, 5, 10, 18, and 19). After applying reverse scoring, a higher total score indicates a greater level of alexithymia in participants.

#### 2.3.4 Chinese version of the beck depression inventory-II, BDI-II-C

This study employed the Chinese version of the Beck Depression Inventory-II (BDI-II-C), which was translated and revised by Yang et al. (2014). This scale is used to assess the severity of depressive symptoms over the past 2 weeks. It contains 21 items, each item was scored at 0 to 3 levels, and the total score of the scale is the sum of 21 items, with the total score range from 0 to 63 points. According to the demarcation points provided by the original scale of Beck et al. (1961), the total score of 0-13 indicates no depression, 14-19 indicates mild depression, 20-28 indicates moderate depression, and 29-63 indicates severe depression.

### 2.4 Data analysis

In this study, data analysis was conducted using SPSS 27.0 for common method bias test, descriptive statistics and correlation analysis. Additionally, the chain mediation model analysis was performed using the SPSS macro PROCESS Model 6.

## 3 Results

### 3.1 Common method bias test

Given that all data in this study were self-reported, the results may be influenced by common method bias. To assess the presence of common method bias, the Harman single-factor test was employed. The results indicated that 17 factors had eigenvalues greater than 1. The variance explained by the first factor was 22.72%, which is below the critical threshold of 40%. Therefore, no significant common method bias was detected (Zhou and Long, 2004).

### 3.2 Construct reliability and validity testing

Confirmatory factor analysis (CFA) was conducted for each scale in this study, and the results indicated a good model fit (Table 2). According to Steiger (1990) recommendation, RMSEA value below 0.1 indicates a good fit, CFI and TLI values greater than 0.8 or 0.9 reflect a satisfactory fit (Hair et al., 2010). Therefore, the measurements of perceived parental rejection, rumination, alexithymia and depression demonstrated adequate construct validity.

**Table 2:** Confirmatory factor analysis.

**Variable**	**RMSEA**	**CFI**	**TLI**	**SRMR**
Mother rejection	0.06	0.99	0.98	0.02
Father rejection	0.04	0.99	0.99	0.02
Rumination	0.08	0.86	0.85	0.05
Alexithymia	0.08	0.82	0.83	0.05
Depression	0.06	0.90	0.89	0.04

Reliability reflects the extent of random error in the measurement and is commonly assessed using Cronbach's α, Composite Reliability (CR), and Average Variance Extracted (AVE) (Chen et al., 2025a). Theoretically, the threshold for factor loading should be >0.5 (Fornell and Larcker, 1981), the threshold for α should be >0.7 (Tavakol and Dennick, 2011), the threshold for CR should be >0.70 (Kahn, 2006), and the threshold for AVE should be >0.5 (Hu and Bentler, 1999). As shown in Table 3, all factor loadings, α and CR values exceed the standard thresholds. Except that the AVE value of depression is close to 0.5, the values of other variables are all above 0.5. However, an AVE value higher than 0.4 still indicates significant statistical significance (Zhou et al., 2023; Chen et al., 2025b), and thus meets the acceptable threshold. This implies that the scale structure constructed in this study has good reliability and validity.

**Table 3:** Construct reliability and validity results.

**Variable**	**Factor loading**	**α**	**CR**	**AVE**
Mother rejection	0.722–0.778	0.851	0.890	0.575
Father rejection	0.728–0.793	0.838	0.882	0.554
Rumination	0.617–0.802	0.940	0.950	0.500
Alexithymia	0.639–0.827	0.846	0.935	0.507
Depression	0.534–0.714	0.913	0.925	0.410

### 3.3 Descriptive statistics and correlation analysis

This study identified that 963 participants exhibited varying levels of depression. The scores of parental rejection, rumination, and alexithymia in the severe depression group were significantly higher than those in the moderate depression group (*p* < 0.001). Similarly, the scores in the moderate depression group were significantly higher than those in the mild depression group (*p* < 0.001). The detailed results are presented in Table 4.

**Table 4:** Comparison of different degrees of depression.

**Degrees of depression**	***N***	**Parental rejection**	**Rumination**	**Alexithymia**
Mild depression	440	8.32 ± 2.33	49.77 ± 10.66	59.34 ± 8.24
Moderate depression	356	9.19 ± 2.91	55.59 ± 10.41	62.39 ± 7.79
Severe depression	167	11.31 ± 3.97	65.50 ± 12.43	65.65 ± 10.19
*F*		168.76	472.95	221.66
*p*		< 0.001	< 0.001	< 0.001

Table 5 demonstrated that there were positive correlations between perceived parental rejection, rumination, alexithymia and depression (all *p* < 0.01). It is worth noting that the high correlation between rumination and depression may be caused by similar items in the two scales. To address this concern, an additional correlation analysis was conducted between the three dimensions of rumination and other variables. The results showed that all three dimensions of rumination had significant positive correlations with other variables, indicating that the similarity between the two scales had no effect on the results (Li et al., 2023).

**Table 5:** Descriptive statistics and correlation analysis (*N* = 2310).

**Variable**	***M***	***SD***	**1**	**2**	**3**	**4**	**5**	**6**	**7**	**8**	**9**
Gender	1.48	0.50	1								
Age	15.88	0.86	−0.03	1							
Parental rejection	8.15	2.59	0.03	0.08^***^	1						
Rumination	45.83	13.44	0.10^***^	0.11^***^	0.39^***^	1					
Symptomatic ruminative	24.06	7.87	0.11^***^	0.12^***^	0.43^***^	0.96^***^	1				
Compulsion thinking	11.50	3.59	0.09^***^	0.09^***^	0.29^***^	0.90^***^	0.80^***^	1			
Introspection thinking	10.27	3.24	0.05^*^	0.08^***^	0.26^***^	0.81^***^	0.66^***^	0.69^***^	1		
Alexithymia	55.64	11.05	0.09^***^	0.04^*^	0.34^***^	0.51^***^	0.57^***^	0.42^***^	0.25^***^	1	
Depression	13.24	9.62	0.16^***^	0.09^***^	0.45^***^	0.67^***^	0.72^***^	0.55^***^	0.43^***^	0.53^***^	1

^*^p < 0.05, ^***^p < 0.001. We use “^*^” to indicate a statistically significant relationship (*p* < 0.05) between variables.

### 3.4 Chain mediating effect analysis

All variables were standardized before conducting data analysis. In order to control the influence of other factors, gender and age were included as control variables. The results of the multiple regression analysis indicated that perceived parental rejection positively predicted rumination (β = 0.38, *p* < 0.001). When both parental rejection and rumination were included as predictive variables, they positively predicted alexithymia (β = 0.17, *p* < 0.001; β = 0.44, *p* < 0.001). When perceived parental rejection, rumination and alexithymia were taken as predictive variables simultaneously, all three variables could positively predict depression (β = 0.19, *p* < 0.01; β = 0.48, *p* < 0.001; β = 0.22, *p* < 0.001) (Table 6).

**Table 6:** Multiple regression analysis.

**Result variable**	**Prediction variable**	***F***	***R***	***R^*2*^***	**β**	***t***
Rumination	Gender	152.71^***^	0.41	0.17	0.09	4.56^***^
	Age				0.08	4.40^***^
	Parental rejection				0.38	19.88^***^
Alexithymia	Gender	226.78^***^	0.53	0.28	0.04	2.03^*^
	Age				−0.02	−1.11
	Parental rejection				0.17	8.95^***^
	Rumination	0.44	22.67^***^
Depression	Gender	522.90^***^	0.73	0.53	0.09	6.18^***^
	Age				0.02	1.28
	Parental rejection				0.19	11.81^***^
	Rumination				0.48	27.60^***^
	Alexithymia				0.22	12.79^***^

^*^p < 0.05, ^***^p < 0.001.

**Figure 2:**
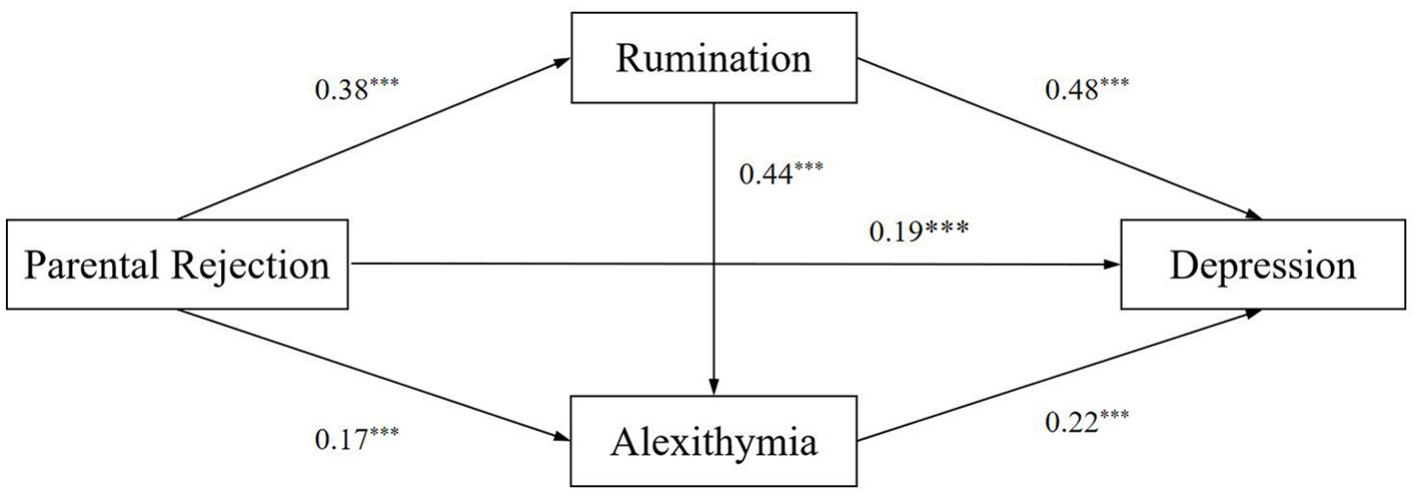
The chain mediating model.

Furthermore, based on the structural equation, the SPSS macro PROCESS Model 6 with 5,000 resamples was used to examine the chain mediation effect of parental rejection and depression. As shown in Table 7, analyses of total indirect effects indicated that rumination and alexithymia served as partial mediating function in the relationship between perceived parental rejection and depression [Effect = 0.25, 95%CI (0.23,0.28)], accounting for 57.66% of the total effect. Meanwhile, when tested separately, all three mediation paths were significant. The indirect effects of perceived parental rejection on depression through rumination [Effect = 0.18, 95%CI (0.16,0.21)], accounting for 41.15% of the total effect. The indirect effects of perceived parental rejection on depression through alexithymia [Effect = 0.04, 95%CI (0.03,0.04)], accounting for 8.14% of the total effect. The indirect effects of perceived parental rejection on depression through rumination and then alexithymia [Effect = 0.04, 95%CI (0.03,0.05)], accounting for 8.39% of the total effect. The specific path of the chain mediation pattern is shown in Figure 2.

**Table 7:** Chain mediating effect test.

**Path**	**Effect**	**SE**	**95%CI**	**Percentage (%)**
Direct effect	0.19	0.02	[0.16, 0.22]	42.34
Indirect effect	0.25	0.01	[0.23, 0.28]	57.66
M1	0.18	0.01	[0.16, 0.21]	41.15
M2	0.04	0.00	[0.03, 0.04]	8.14
M3	0.04	0.00	[0.03, 0.05]	8.39
Total effect	0.44	0.02	[0.40, 0.45]	

## 4 Discussion

The detection rate of severe depression among high school students in this study was 7.23%, which aligns with the high prevalence trend of depression among adolescents globally in recent years. For instance, a 2021 meta-analysis published in the Journal of the American Medical Association that included data from 44 studies reported a weighted average detection rate of major depressive disorder in adolescents worldwide of 6.2% (95% CI: 5.2%-7.3%) (Racine et al., 2021), a figure comparable to the results of this study. Additionally, research in China supports this trend. The Report on the Development of China's National Mental Health (2019-2020), released by the Institute of Psychology at the Chinese Academy of Sciences, indicated that the overall detection rate of depression among Chinese adolescents was 24.6%, with 7.4% representing severe depression. Furthermore, the data revealed that levels of parental rejection, rumination, and alexithymia increased with the severity of depression, consistent with previous findings (Yu et al., 2024; Guo et al., 2024; Sagar et al., 2021).

### 4.1 The relationship between perceived parental rejection and depression

The results of this study indicate a positive relationship between perceived parental rejection and depression, which supports hypothesis 1 and aligns with previous research findings (Yu et al., 2024; Peng et al., 2021; Christ et al., 2017). According to parental acceptance-rejection theory, the core of parental rejection lies in children's emotional deficiency. This parenting style is associated with persistent adverse outcomes in the psychological development of children and adolescents, and is accompanied by negative self-perceptions and worldview, with individuals experiencing higher levels of parental rejection also showing elevated depression risk (Ramírez-Uclés et al., 2018). Furthermore, adolescents who perceive parental rejection demonstrate heightened susceptibility to maladaptive behaviors. The perception of such rejection is linked to feelings of isolation and exclusion, which are often associated with emotional alienation and loneliness. Concurrently, the perception of parental rejection is correlated with an internal attributional cognitive pattern, which typically manifests as social withdrawal tendencies and diminished self-efficacy in subsequent behaviors. The perpetuation of this psychological state is often associated with disruptions in emotional regulation systems, and is thus linked to increased incidence of depression, anxiety, and other negative affective states in youth.

This finding further substantiates the core tenet of Bowen (1993) Family Systems Theory, providing insights into the associative mechanism between negative parenting styles and adolescent psychological development. Negative parenting behaviors (e.g., emotional indifference, excessive punishment and rejection) are associated with weakened family functions in multiple dimensions. Primarily, these behaviors are linked to difficulties in establishing and maintaining a supportive familial atmosphere, and are often accompanied by significant weakening of the emotional bonds between parents and children. Secondly, such parenting models are correlated with substantially elevated stress levels of adolescents, which in turn are associated with cumulative growth in negative emotional experiences. Thirdly, adolescents in such environments demonstrate diminished psychological resilience over time. When confronting daily stressors, these individuals typically exhibit low self-efficacy and maladaptive cognitive-behavioral patterns, which are in turn correlated with heightened depression risk.

### 4.2 The mediating role of rumination

This study highlights the mediating role of rumination in the relationship between perceived parental rejection and depression, thereby confirming Hypothesis 2. Firstly, perceived parental rejection positively predicts rumination, consistent with previous research findings that adolescents who perceive more parental rejection are more likely to fall into rumination (Meng et al., 2024). As an interpersonal stressor, parental rejection makes adolescents feel emotional indifference, insecurity, and a sense of incompetence within the family, which can trigger intense negative emotional reactions and may also lead to deficits in emotional regulation (Sobrinho et al., 2016). Impaired emotional regulation makes it difficult for individuals to effectively manage and regulate their emotions in the face of challenges and difficulties, potentially triggering recurrent intrusive thoughts about negative events. Specifically, individuals with such impairments tend to be trapped in a passive, repetitive focus on distress symptoms and their potential causes and consequences, ultimately establishing a state of rumination (Watkins and Roberts, 2020).

Additionally, rumination positively predicts the level of depression, which is consistent with previous research findings indicating that rumination is a significant predictor of depression (Tong and Jiang, 2023). According to the response styles theory, rumination is correlated with the exacerbation and prolongation of negative emotional experiences, and is associated with impairment of social cognitive functioning. This impairment manifests as diminished social comprehension, unrealistically high social expectations, and heightened disillusionment with life (Nolen-Hoeksema et al., 2008). As a maladaptive emotion regulation strategy, rumination not only shows no association with effective problem-solving (Michl et al., 2013), but also correlates with the perpetuation of negative states through continuous depletion of cognitive resources. This pattern of rumination is linked to intensified feelings of hopelessness, helplessness, worthlessness, sadness, and other depressive symptoms (Hilt et al., 2008). Therefore, rumination plays a mediating role in the relationship between perceived parental rejection and depression.

### 4.3 The mediating role of alexithymia

This study also found that alexithymia mediates the relationship between perceived parental rejection and depression, thereby supporting Hypothesis 3. Parental rejection positively predicts alexithymia in adolescents, which aligns with the findings of Fabris et al. (2025). The occurrence of alexithymia is closely associated with factors such as family function and environment (Zhang et al., 2011). During adolescence, individuals typically exhibit heightened self-attention and self-awareness. In the course of psychological development, they experience a contradictory state characterized by both a desire to break free from parental protection and supervision in pursuit of independence and autonomy, and a concurrent need to maintain an emotional bond with their parents (Guo and Lei, 2006). When parents dismiss their children's emotional appeals and ideological expressions, they often impose irrational and excessive punishment while exhibiting emotional detachment. Simultaneously, they exert overprotection and behavioral control in daily life management. This parenting style results in teenagers failing to develop effective emotional discrimination and expression skills, thereby fostering a self-isolated state. Such a state is specifically characterized by difficulties in self-perception recognition, emotional expression disorders, and a lack of adaptive emotional communication abilities (Lu et al., 2021).

In addition, the study found that alexithymia positively predicts depression in adolescents. Research has indicated that alexithymia can exert multi-dimensional negative effects on individual emotions (Chen, 2016), interpersonal relationships (Xue et al., 2021), and behaviors (Fanton et al., 2022). Alexithymia is characterized by impairments in emotional processing and regulation. Due to their inability to implement effective emotion regulation strategies, individuals with alexithymia demonstrate elevated susceptibility to psychological disorders such as depression (Preece et al., 2022; Qian et al., 2020). Consistent with affective exchange theory, individuals with high alexithymia levels experience difficulty engaging in emotionally intimate and meaningful interpersonal communication (Ren et al., 2022). Their characteristic manifestations include distorted emotional self-expression and biased perception of others' emotions. Consequently, when confronting negative social evaluations or rejection, they exhibit heightened vulnerability to loneliness. The fear of social rejection combined with perceived helplessness in managing social challenges ultimately increase the risk of depression. Thus, alexithymia serves as a mediator in the relationship between perceived parental rejection and depression.

### 4.4 Chain mediating role of rumination and alexithymia

The present study further found that rumination and alexithymia chain mediate the effects of perceived parental rejection on depression, thereby supporting Hypothesis 4. This finding aligns with previous research demonstrating a strong correlation between rumination and alexithymia, where rumination positively predicts alexithymia (Luo et al., 2021). Rumination promotes the formation of an inward-focused cognitive style, characterized by excessive self-attention and diminished observation of external environments and social interactions. This progressive cognitive narrowing is associated with progressive impairments in emotional recognition and expression capabilities, and such impairments are concurrently linked to the development of alexithymia. On the other hand, adolescents with high rumination are more likely to develop maladaptive repetitive negative cognitive patterns when processing emotional stimuli. Paradoxically, they believe such repetitive thinking helps them gain insight into coping with emotional challenges. However, these persistent negative cognitions prompt high rumination individuals to increasingly employ emotion regulation strategies that suppress emotional expression (Di Schiena et al., 2011). This emotion regulation strategy is not only unassociated with the alleviation of negative emotional experiences, but also correlates with greater physiological activation, forming a vicious cycle in emotion regulation that is linked to an increase in alexithymia symptoms (Dan-Glauser and Gross, 2011). Accordingly, rumination and alexithymia play a chain mediating role in the relationship between perceived parental rejection on depression.

Overall, parental rejection parenting styles lack warmth, respect, and support toward high school students. This deprivation triggers feelings of emotional isolation and an impaired sense of belonging, prompting adolescents to repeatedly ruminate about rejection causes while becoming immersed in negative affect. Chronic rumination fosters an internalizing pattern of emotion processing rather than adaptive externalizing strategies. Consequently, this diminishes emotional identification, expression, and regulation capacities, ultimately increasing vulnerability to depression.

## 5 Limitations and perspectives

There are certain limitations in this study that warrant attention and resolution in future research. Firstly, this study examines parents as a collective group, which may obscure the mechanisms underlying individual differences among parents. Given substantial differences in parenting style intensity and influence pathways between fathers and mothers (Meng et al., 2024), subsequent studies could employ hierarchical regression modeling to separately test the independent effects of paternal and maternal rejection, as well as explore their potential moderating effects through interaction term analysis. Secondly, previous studies have found that children who frequently engage in rumination about problems may be more prone to perceiving parental rejection (Cheah et al., 2019). Studies on trait alexithymia have shown that individuals are more likely to perceive parental rejection due to alexithymia. Therefore, there may be complex interactions or bidirectional influences among the variables. However, this study only preliminarily explored the unidirectional relationship of the model based on theoretical assumptions. Consequently, future research could incorporate longitudinal or experimental designs to elucidate the causal relationships among these variables. Thirdly, there are numerous potential confounding variables which were never examined such as trait alexithymia, initial depressive symptoms etc which could also impact the model. Future research needs to adopt longitudinal designs and control for these variables in order to conduct validation. Finally, this study primarily relied on self-reports from adolescents. Future research could incorporate parent reports or observational data to differentiate between the effects of perceived rejection and actual parental rejection behaviors, thereby providing deeper insights into the underlying.

## 6 Educational Suggestion

This study elucidates the intricate chain-mediated relationship between parental rejection and depression, providing both a theoretical foundation and practical guidance for intervening in depression among high school students.

First and foremost, as pivotal figures in adolescents' development, parents must deeply comprehend the potential detrimental effects of rejection behaviors (e.g., emotional indifference, neglect, and derogatory remarks) on adolescents' psychological growth. In everyday life, parents should learn to replace rejecting behaviors with responsive support. It is essential for parents to proactively attend to the emotional needs and developmental dynamics of high school students. They should respond promptly through active listening, empathy, physical affection, and other supportive measures. Additionally, a problem-solving communication model should be established. When adolescents face setbacks, parents should first listen to their emotional expressions before jointly exploring potential solutions. Schools can also implement parent-child emotional communication trainings to provide parents with ongoing guidance, ensuring that adolescents receive consistent cognitive regulation support both at home and in school. This approach helps high school students build secure attachment relationships and cultivate positive self-identity.

Secondly, to break the chain by which persistent rumination exacerbates deficits in emotional awareness, schools can integrate cognitive restructuring and emotional awareness training to develop structured group intervention programs. In cognitive restructuring training, teachers should help students deconstruct cognitive biases and guide them to shift from an “immersion-rumination” mode to an “analysis-solution” mode. This involves making multi-dimensional attributions for setback events (self, others, environment) and focusing on at least one controllable improvement action. Immediately following cognitive restructuring, emotional awareness exercises should be conducted to help adolescents translate the restructured cognition into specific emotional vocabulary and bodily sensations, thereby simultaneously weakening the two key transmission pathways of the chain mediation and achieving more effective prevention outcomes. Additionally, incorporating mindfulness training can also help students objectively evaluate emotional experiences and reshape maladaptive cognitive-affective patterns (Yuan et al., 2025).

Finally, to mitigate the risk of depression caused by impaired emotional expression, we focus on contextualized emotion regulation training. For families and schools, it is essential to construct a “safe emotional expression ecosystem,” encouraging adolescents to communicate their feelings in a non-judgmental environment and training them to refine their emotional expression abilities, thereby blocking the pathway leading to depression. Techniques such as emotion recognition training and guidance on emotional expression methods can help them understand the mechanisms underlying emotion generation. At the same time, it is essential to establish an open and inclusive communication environment within families. This encourages high school students to freely express their emotional experiences, thereby promoting the development of emotional regulation skills. Such approaches effectively alleviate emotional expression barriers and reduce susceptibility to depression (Xu et al., 2024).

## 7 Conclusions

To explore the intrinsic mechanisms linking perceived parental rejection and depression, this study constructed a chain mediation model. The research validated a cognitive-emotional dual-channel model for the transmission of familial emotional risk to psychopathological symptoms, revealing the chain mediating role of rumination and alexithymia between perceived parental rejection and depression. It also provides empirical support for family systems theory, parental acceptance-rejection theory, and response styles theory. The findings indicate that parental rejection not only directly elevates depression risk, but also indirectly influences depression through rumination and alexithymia chain mediation. Parental rejection disrupts individuals' cognitive processing of negative experiences, leading to persistent rumination on painful memories. Such impairment diminishes metacognitive abilities related to emotion recognition and expression, thereby increasing susceptibility to depression.

## Data Availability

The raw data supporting the conclusions of this article will be made available by the authors, without undue reservation.
